# Systematic reviews of ten pharmaceutical pricing policies – a research protocol

**DOI:** 10.1186/s40545-020-00228-0

**Published:** 2020-07-16

**Authors:** David Tordrup, Hendrika A. van den Ham, Julie Glanville, Aukje K. Mantel-Teeuwisse

**Affiliations:** 1 Utrecht Centre for Pharmaceutical Policy and Regulation, Division of Pharmacoepidemiology & Clinical Pharmacology, Utrecht Institute for Pharmaceutical Sciences (UIPS), Utrecht University, Utrecht, the Netherlands; 2 York Health Economics Consortium, York, UK

**Keywords:** Pharmaceutical pricing policies, Protocol, Systematic literature review, GRADE, Risk of bias

## Abstract

**Background:**

High prices of pharmaceutical products are an increasing challenge in high- and low-income countries. Governments in many countries have implemented pricing policies to ensure affordability of medicines to patients and healthcare systems. The World Health Organization published in 2015 the Guideline on Country Pharmaceutical Pricing Policies, which was based on a series of evidence reviews in the preceding years.

As part of the ongoing update of this guideline, we present a protocol for 10 systematic literature reviews on pharmaceutical pricing policies to be covered by the updated guideline.

**Methods:**

The systematic literature reviews will be undertaken according to the principles embodied in the Cochrane Handbook and Centre for Reviews and Dissemination. The interventions studied are pharmaceutical pricing policies implemented by public institutions or a group of purchasing organizations/individuals (e.g. health services). Studies reporting price, volume, availability and/or affordability as the primary outcomes will be eligible for inclusion. Studies in any country or jurisdiction, in any language and in any setting published in 2004 or later are eligible. Eligible study designs are randomized and non-randomized trials, and observational studies including cohort studies, panel data analyses, comparative time series design (including interrupted time-series and repeated measures studies), and controlled before-after studies. A list of 21 databases of peer-reviewed and grey literature will be searched, along with supplementary searches of relevant national and international organizational and governmental websites. Risk of bias will be assessed according to the Cochrane Effective Practice and Organisation of Care (EPOC) guidelines. A summary table according to the EPOC Worksheets for preparing a Summary of Findings table (SoF) using GRADE will be provided.

**Discussion:**

The results of the review will be used as part of the update of the WHO Guideline on Country Pharmaceutical Pricing Policies. The current protocol may serve as an example for performing systematic literature reviews to inform policy makers.

## Background

In recent years, high prices of pharmaceutical products have posed challenges in high- and low-income countries alike. In many instances, high prices of pharmaceutical products have led to significant financial hardship for individuals and negatively impacted on healthcare systems’ ability to provide population-wide access to essential medicines [1]. Governments in many countries have implemented pricing policies to ensure affordability of medicines to patients and healthcare systems.

In view of these problems and the overall mission of the World Health Organization (WHO), WHO has mandates to support countries in ensuring that medicines are affordable, by providing policy guidance on pricing of pharmaceutical products, as requested by Member States. These mandates include World Health Assembly (WHA) decision WHA71(8), which requested the Director-General to elaborate a road map report outlining the programming of WHO’s work on access to medicines and vaccines for the period 2019–2023. Guidance on pricing policy is one of the key milestones specified in this road map [2]. The WHO 13th General Programme of Work includes various areas of work relating to pricing to improve access to medicines, vaccines and health products such as fair pricing, and WHO Regional Committees Resolutions relating to access to medicines have noted the importance of having robust policies on the pricing of health products [3]. Finally the United Nations (UN) Sustainable Development Goals (SDGs) recognise the importance of achieving universal health coverage, through “financial risk protection, access to quality essential health-care services and access to safe, effective, quality and affordable essential medicines and vaccines for all” (SDG 3.8) [4].

In 2015, WHO published a guideline on country pharmaceutical pricing policies (“2015 Guideline”) [5]. This guideline was developed based on an evidence review conducted in 2010 and will be updated in 2020. Since 2010, the body of literature documenting the effects of various government pricing policies has increased. To ensure the updated guideline recommendations reflect current evidence base, WHO has commissioned this systematic review to provide an updated synthesis of the relevant evidence for the existing guideline, and to answer additional policy questions relating to pricing of pharmaceutical products.

The overall objective of this review is to assess the effects of 10 pharmaceutical pricing policies implemented individually or in combination, by an institution or a group of organizations, on price, volume, availability, and affordability of pharmaceutical products.

The specific objectives are (1) to the extent possible, estimate the effect size of pricing policy or policies on each prespecified outcome, (2) to the extent possible, develop Grade of Recommendation, Assessment, Development and Evaluation (GRADE) evidence profiles for each research question, with a view to assessing the overall strength, direction and quality of the evidence, and (3) to the extent possible, describe any practical and contextual considerations, based on an assessment of qualitative evidence pertaining to countries’ experiences, that might impact the implementation of pricing policies.

## Methods

The systematic reviews will be undertaken according to the principles of systematic reviewing embodied in the Cochrane Handbook and guidance document published by the Centre for Reviews and Dissemination (CRD) which offers approaches for a range of study designs beyond randomised clinical trials (RCTs) [6, 7].

### Types of interventions

The interventions are pharmaceutical pricing policies, which are defined as a set of written principles or requirements agreed or adopted by a public institution (e.g. a government) or a group of purchasing organizations/individuals (e.g. health services) for managing the prices of pharmaceutical products for human use. A pharmaceutical product, commonly referred interchangeably with drug, medicine or pharmaceutical, is defined as any manufactured or refined substance for human or veterinary use that is intended to modify or explore physiological systems or pathological states for the benefit of the recipient (adapted from WHO Glossary [8]). For the purpose of this review, the scope includes medicines (both small molecules and biological products) and vaccines for human use. There are 10 eligible policies or strategies within the scope of this review, as defined in Table 1. Single policies, or combinations of policies, are considered eligible. The review scope includes studies comparing interventions against other interventions, options or strategies, or a counterfactual in the absence of comparator interventions, including historical comparisons. Studies not describing any comparator or counterfactual will not be eligible for inclusion in the reviews.

**Table 1:** Definitions of pricing policies and strategies

Term	Definition (from [9] unless otherwise stated)
Reference pricing	Reference pricing, also known as benchmark pricing, refers to the approach of understanding the appropriateness of prices of medicines based on selected benchmark prices, either from other jurisdictions (e.g. countries or other administrative regions) or a group of comparable medicines in the same system/formulary. For the purpose of this review, reference pricing is stratified as external and internal reference pricing: External reference pricing (ERP; also known as international reference pricing) refers to the practice of using the price of a pharmaceutical product (generally ex-manufacturer price, or other common point within the distribution chain) in one or several countries to derive a benchmark or reference price for the purposes of setting or negotiating the price of the product in a given country. Reference may be made to single-source or multisource supply products [5] The practice of using the prices of identical medicines (ATC 5 level) or similar products (ATC 4 level) or even with therapeutic equivalent treatment (not necessarily a medicine) in a country in order to derive a benchmark or reference price for the purposes of setting or negotiating the price or reimbursement of the product in a given country.
Value-based pricing	Countries set prices for new medicines and/or decide on reimbursement based on the therapeutic value the medicines confer, usually assessed through health technology assessment (HTA).
Cost-plus pricing	Pricing policy that takes into account production costs, promotional expenses, research & development, administration costs, overheads and a profit to determine a price.
Setting price and mark-up thresholds across the pharmaceutical supply and distribution chain	Setting price threshold means specifying maximum prices, also referred to as price caps or price ceilings, or specifying maximum mark-up percentage. A mark-up represents the additional charges and costs that are applied to the price of a commodity in order to cover overhead costs, distribution charges, and profit. In the context of the pharmaceutical supply chain, policies might involve regulation of wholesale and retail mark-ups as well as pharmaceutical remuneration.
Promoting price transparency	The sharing, disclosure and dissemination of information related to medicine prices to the public and relevant parties to ensure accountability. Full price transparency includes the publication of medicine prices at all price types (e.g. ex-factory prices, pharmacy retail prices), the disclosure of the net transaction prices of medicines between the suppliers (e.g. manufacturers, service providers) and the payers/purchasers (governments, consumers), the sharing and publication of the contents of pricing arrangements, such as risk-sharing schemes and other managed-entry agreements, including the actual pricing and input factors that determine a medicines prices (e.g. production costs, R&D costs, added therapeutic value). (adapted from [10])
Price discounts for single source pharmaceuticals	Discount is the general term to describe to a price reduction granted to specified purchasers under specific conditions prior to purchase. Different types of price reductions include a rebate (payment made to the purchaser after the transaction has occurred), or upon meeting certain pre-agreed terms and conditions as specified in so-called managed-entry agreements. The latter arrangements are usually classified into financial-based MEA (e.g. flat discounts, price-volume agreements, capping) and performance-based MEA (e.g. risk-sharing agreement, coverage with evidence development). Single source pharmaceuticals are pharmaceutical products supplied by a company that holds the patent rights, exclusive marketing rights, or supply agreements in a specific jurisdiction.
Promoting the use of quality assured generic and biosimilar medicines	Strategies directed at patients, prescribers or pharmacists to encourage the use of generic or similar biological medicines.
Competitive pricing based on tendering and negotiation	An approach that determines prices through tendering or negotiation among suppliers of medicines that are identical or comparable in chemical composition, pharmacological mechanisms and therapeutic use, taking into account factors such as quality, supply conditions. Tendering is any formal and competitive procurement procedure through which tenders (offers) are requested, received and evaluated for the procurement of goods, works or services, and as a consequence of which an award is made to the tenderer whose tender/offer is the most advantageous. Negotiation refers to “discussion aimed at reaching an agreement” [11]
Pooled procurement	Pooled procurement refers to the arrangement where financial and non-financial resources are combined across various purchasing authorities to create a single entity for purchasing health products (e.g. medicines) on behalf of the individual purchasing authorities [10]
Tax exemptions or tax reductions for pharmaceuticals	Tax is a compulsory transfer of money from private individuals, institutions or groups to the government. It may be levied upon wealth or income (direct taxation) or in the form of surcharges on prices (indirect taxation). It may be paid to the central government (central taxation) or to the local government (local taxation). There are two main categories of tax: direct taxes, which are levied by governments on the income of individuals and corporations, and indirect taxes, which are added to the prices of goods and services. Direct taxes, along with social security taxes, generally make up about two-thirds of total government revenue in high-income countries. In low-income countries, indirect taxes, on international trade or on the purchase of goods and services, are major sources of government revenue. Policies relevant to pharmaceutical products might involve the reduction of taxes on medicines, or the exemption of medicines from taxes, particularly sales taxes [5]

### Types of outcome measures

Studies reporting price, volume, availability and/or affordability as primary outcomes will be eligible for inclusion in the systematic reviews. Studies that report the primary outcomes will also be assessed for information on any secondary outcome, including transparency, system efficiencies, and adverse outcomes (shortages, quality issues, safety issues, unethical conduct, illegal conduct, equity). Definitions of primary and secondary outcomes are provided in Tables 2 and 3, respectively.

**Table 2:** Definitions of the primary outcomes

Term	Operational definition	Measurement unit
Price	Price components, observed or derived, along the value chain from manufacturer, distributor, service providers to patients	Absolute or percentage changes in reported currency unit(s) or price indices. Expenditure or sales data (aggregate of price and volume) as a proxy for price and volume if these are not individually reported.
Volume	Quantity provided or used	Number of units sold, supplied, prescribed, dispensed, or consumed
Availability	A patient is able to obtain when needed, for free or for a fixed fee, a pharmaceutical product which is listed on the national formulary	Presence-absence binary measurement and qualitative assessment as reported, e.g. a medicine is available when it is found in this facility by the data collector on the day of the visit
Affordability	“the ability to purchase a necessary quantity of a product or level of a service without suffering undue financial hardship” World Bank cited by Lancet Commission on Essential Medicines.	For health system: Proportion of spending on medicines compared to historical expenditure on medicines or other health products and services, or as reported in the literature
		For individual patients: The number of days’ wages needed to pay for the cost of treatment, using wage benchmarks such as salary of the lowest paid government worker and national minimum wage, or as reported in the literature

**Table 3:** Definitions of secondary outcomes

Term	Operational definition	Measurement unit
Transparency	See price transparency in Table 1.	Qualitative description, as presented in literature
Efficiency	AHRQ’s definition: Avoiding waste, including waste of equipment, supplies, ideas, and energy. Allocative efficiency: Allocating resources in such a way as to provide the optimal mix of goods and services to maximise the benefits to society Technical efficiency: Using the least amount of resources or the right combination of inputs to produce a given mix of goods and services	As measured and presented in literature or qualitative description, as presented in literature Qualitative measures of process efficiency, e.g. timeliness, resource-intensiveness
Shortage	EMA’s definition: a shortage of a medicinal product occurs when there are changes to either demand or supply of the medicine, so that clinical need can no longer be met. A medicine shortage causes temporary unavailability. The total stock across all levels of the national supply chain, across all geographical regions, cannot meet demand during a medicine shortage	As measured and presented in literature or qualitative description, as presented in literature
Quality of pharmaceutical products	Whether products are substandard or falsified (SF) WHO’s definitions: Substandard: Also called “out of specification”, these are authorized medical products that fail to meet either *internationally accepted* quality standards or specifications, or both. Falsified: Medical products that deliberately/fraudulently misrepresent their identity, composition or source.	Occurrence of SF products
Safety	IOM’s definition: the prevention of harm to patients	As measured and presented in literature
Unethical conduct	Business or professional conduct that contravenes social norms or social responsibilities	Qualitative description, as presented in literature
Illegal conduct	Business or professional conduct that contravenes the law	Qualitative description, as presented in literature
Equity	Differences in [access or] health that are avoidable and also considered unfair or unjust	Qualitative assessment, including assessing differences in the relative effect size of the intervention; assessing indirectness of evidence to disadvantaged populations and/or settings.

AHRQ: Agency for Healthcare Research and Quality, EMA: European Medicines Agency, IOM: Institute of Medicine, WHO: World Health Organization

### Contextual factors

Descriptions of the contextual factors or prerequisites (barriers or facilitators) in included studies, which have contributed to the success or failure of a policy intervention, will be identified and extracted. This includes strategies that were used to implement an intervention successfully. Studies reporting descriptive and qualitative information on the implementation of effective/successful interventions, in addition to the primary outcome(s), will be eligible.

### Study settings

Studies conducted in any country or other jurisdictions (e.g. administrative regions) will be eligible. A subgroup of interest is studies that focus on middle and low-income countries. Outcomes in public, private and mixed public-private settings are of interest.

### Study designs

The following study designs comparing interventions to at least one comparator or counterfactual are eligible:

- randomized trial
- non-randomized trial
- observational studies, including:
  - cohort studies or panel data analysis
  - comparative time series design, including interrupted time series (ITS) and repeated measures (RM) study
  - controlled before-after study

Existing systematic reviews on relevant pharmaceutical pricing policies will not be included in the present review due to potential differences in scope and methodology, but any reviews identified will be discussed in the present reviews, and references from recent reviews (published in the last 5 years) will be checked for eligibility. A summary of all inclusion and exclusion criteria is provided in Table 4.

**Table 4:** Summary of the inclusion and exclusion criteria

	Inclusion criteria	Exclusion criteria
Intervention	Ten pharmaceutical pricing policy interventions, as specified and defined in Table 1	Studies without one of the ten prespecified policy interventions
Outcome	Studies including price, volume, availability or affordability as primary outcome	Studies without one of the four primary outcomes
Study design	randomised trial, non-randomised trial, and observational studies with at least one comparator or counterfactual	All other study designs that do not include at least one comparator or specifying a counterfactual. These include case series.
Countries	All countries	None
Settings	All settings	None
Time period	2004–2019 (publication date)	Studies with publication date before 1 January 2004
Language	All languages	None

### Search strategy

The resources and supplementary search approaches proposed reflect the topic area and the anticipated importance of grey literature. Literature on pharmaceutical pricing policies has relevance to both healthcare, economics and policy research. A base strategy will be developed in MEDLINE (OvidSP) and translated to equivalent searches in other databases; the final Ovid MEDLINE strategy (Supplementary materials in Annex) will be peer-reviewed by a second Information Specialist for errors in spelling, syntax and line combinations.

The search will include the following databases: Ovid MEDLINE(R) and Epub Ahead of Print, In-Process & Other Non-Indexed Citations and Daily; Embase; Cochrane Database of Systematic Reviews (CDSR); Epistemonikos; Database of Abstracts of Reviews of Effects (DARE); Health Technology Assessment (HTA) Database; Social Science Citation Index; LILACS; EconLit; NHS Economic Evaluation Database (NHS EED); INRUD (International Network for Rational Use of Drugs) Bibliography; OECD iLibrary Books (freely accessible content only); International Political Science Abstracts (IPSA); WHO IRIS (Institutional Repository for Information Sharing); World Bank Documents & Reports; World Bank Open Knowledge Repository; World Bank eLibrary (freely accessible content only); IDEAS; Essential Medicines and Health Products Information Portal (WHO); Open Grey; Global Index Medicus.

Searches will be conducted using each database or resource listed, translating the Ovid MEDLINE strategy appropriately. Translation includes consideration of differences in database interfaces and functionality, in addition to variation in indexing languages and thesauri.

In addition, in the context of the balanced bibliographic database search approach, the main database searches will be complemented by supplementary search approaches: a range of governmental and intergovernmental websites will be searched, and experts (WHO Guideline Development Group, project expert panel) will be contacted for additional reference suggestions.

Since the topic context is one where developing a robust strategy without retrieving large numbers of records is challenging, a number of pragmatic decisions have been taken when developing the strategy, which can be found in Supplementary Material in the Annex.

Where possible, the results of searches will be downloaded in a tagged format and loaded into bibliographic management software (EndNote). The results will be deduplicated using several algorithms and the deduplicated references held in a duplicates EndNote database for checking if required. Results from resources which do not allow export in a format compatible with EndNote will be saved in Word or Excel documents as appropriate and manually deduplicated.

### Study selection

Record assessment involves a number of stages. First, a single researcher will assess the search results according to their relevance in providing information on the review, and will remove the obviously irrelevant records based on titles and abstracts such as those that are about treatment effectiveness rather than pricing policy. This will be undertaken within Endnote. Records will be tagged if excluded at the title/abstract stage. Then, the titles and abstracts of remaining records will be assessed for relevance against the protocol criteria by double independent reviewer selection with disagreements adjudicated by a third reviewer. This will be undertaken within Covidence. Covidence allows record tagging which means at this stage records will be initially categorised by the review question they may be able to inform. Once completed we will go back and tag the EndNote library with the include/exclude decisions and group records within EndNote by exclusion reason. Finally, the full text of potentially relevant studies will be obtained and these will be assessed for eligibility against the protocol criteria by double independent reviewer selection with disagreements adjudicated by a third reviewer. This will be undertaken in Covidence. Studies will be tagged by the review question they will inform, so that they can be grouped for assessment at the next stage. Finally, references of included studies will be screened for further potentially eligible studies.

The number of records included and removed at each stage will be recorded in a PRISMA flow diagram. Studies excluded after assessment of the full document for each review will be listed in an excluded studies table with the reason for exclusion. Where results for a study are reported in more than one publication (for example a full paper and a conference abstract), all related publications will be identified and grouped together to ensure that studies are not double counted.

### Data extraction and risk of bias assessment

Included studies will be extracted using a standard data extraction form, including information on study details, population/setting/subjects, interventions, outcomes and results. One researcher will extract the data, and the extraction will be checked by a second researcher. Risk of bias will be assessed according to the Cochrane Effective Practice and Organisation of Care (EPOC) guidelines [12]. Bias assessment criteria are adapted to study design (randomized-, non-randomized trials and controlled before-after studies assessed in the same way; interrupted time-series and repeated measures studies assessed in the same way; and a set of assessment criteria applied to all study types). Two researchers will assess risk of bias, resolving conflicts through discussion.

### Data synthesis and analysis

We will provide a summary table according to the EPOC Worksheets for preparing a Summary of Findings table (SoF) using GRADE, which will summarise strength of evidence for each primary outcome. Any evidence identified as reviews/synthesis of qualitative evidence will be assessed using GRADE-CERQual [13, 14].

As it is expected that there will be substantial differences in the characteristics and contexts of included studies, we do not plan to undertake a meta-analysis. If several studies report on the same outcome, a mean/median and a range will be given. Otherwise a narrative summary describing the quality of the studies, the relationship between studies, and patterns that we have discerned in the data will be provided.

Where applicable, results will be presented according to subgroups of interest which are studies by type of medicines (medicines listed in National Essential Medicines Lists, high-priced medicines), studies in low- and middle-income countries, and studies in the private, public or mixed sectors.

## Discussion

These 10 different systematic reviews will inform the WHO Guideline Development Group to update the Guidelines for pharmaceutical pricing policies. A strength of the search methodology described is that it is very broad, making use of multiple scientific literature databases, grey literature and including both English and non-English articles. This will result in limited reporting bias. However, because search terms need to cover ten topics and terminology is not always straightforward the search results might result in an unprecise output. This would mean that it is possible that many titles need to be screened at title and abstract level.

From experience the number of good quality studies is low in policy research [15]. Limited or no randomized studies exist and often studies present before and after measurements around an intervention without taking into account any form of control. These studies will be excluded and this possibly results in a limited number of papers that can be used for the review. However, it was decided to only include study designs that can provide conclusions with limited chance of bias, as we believe that evidence-based decision making is of the same importance for policy makers as it is for clinicians.

## Data Availability

Not applicable.
